# Rituximab for non-infectious Uveitis and Scleritis

**DOI:** 10.1186/s12348-021-00252-4

**Published:** 2021-08-16

**Authors:** Caleb C. Ng, Aileen Sy, Emmett T. Cunningham

**Affiliations:** 1 Department of Ophthalmology, California Pacific Medical Center, San Francisco, CA USA; 2 West Coast Retina Medical Group, 1445 Bush Street, San Francisco, CA 94109 USA; 3 Department of Ophthalmology, Kaiser Permanente Santa Clara, California, USA; 4 Department of Ophthalmology, Stanford University School of Medicine, Stanford, CA USA; 5 Francis I. Proctor Foundation, UCSF School of Medicine, San Francisco, CA USA

**Keywords:** Autoimmune retinopathy, Intraocular inflammation, Rituxan

## Abstract

**Purpose:**

To provide a comprehensive review of rituximab use for the treatment of non-infectious uveitis and scleritis.

**Methods:**

Review of literature through December 2020.

**Results:**

Individual data was available for 229 patients with refractory non-infectious uveitis (*n* = 108) or scleritis (*n* = 121) who received treatment with rituximab (RTX). Rituximab was generally utilized as third-line or later treatment (uveitis: 67/90, 74.4%; scleritis: 90/96, 93.8%) at a mean of 33.5 months following the diagnosis of uveitis (range = 0 to 168.0 months; median = 24.0 months) and 39.4 months after diagnosis of scleritis (range = 1.0 to 168.0 months; median = 21.0 months). Patients with non-infectious uveitis and scleritis either received prior treatment with corticosteroids only (uveitis: 18/90, 20%; scleritis: 4/94, 4.3%), or with one (uveitis: 19/90, 21.1%; scleritis: 30/94, 31.9%), two (uveitis: 11/90, 12.2%; scleritis 27/94, 28.7%), or three or more (uveitis: 37/90, 41.1%; scleritis: 31/94, 33.0%) corticosteroid-sparing immunosuppressive agents with or without corticosteroids before initiation of RTX treatment. The rheumatologic protocol (two infusions of 1 gram of RTX separated by 14 days) was utilized most frequently (uveitis: 45/87, 51.7%; scleritis: 87/114, 76.3%), followed by the Foster protocol (eight weekly infusions of 375 mg/m^2^ RTX; uveitis: 18/87, 20.7%; scleritis: 10/114, 8.8%), and the oncologic protocol (four weekly infusions of 375 mg/m^2^ RTX; uveitis: 5/87, 5.7%; scleritis: 6/114, 5.3%). Various other off-label regimens were used infrequently (uveitis: 19/87, 21.8%; scleritis 11/114, 9.6%). Rituximab treatments resulted in a positive therapeutic response for the majority of patients with non-infectious uveitis (81/97, 83.5%). Commonly treated uveitic diagnoses included non-paraneoplastic autoimmune retinopathy (30/107, 28.0%), juvenile idiopathic arthritis (21/107, 19.6%), Vogt-Koyanagi-Harada disease (12/107, 11.2%), and Behçet disease (11/107, 10.3%). Cases of non-infectious scleritis were most commonly attributed to granulomatosis with polyangiitis (75/121, 62.0%) and rheumatoid arthritis (15/121, 12.4%), and showed an even greater rate of positive therapeutic response (112/120, 93.3%) following RTX treatment. No side effects were reported in 76.3% (74/97) of uveitis and 85.5% (71/83) scleritis cases. Of those cases associated with RTX-induced adverse events, the most common were infusion reactions of various severity (11/35, 31.4%).

**Conclusions:**

Overall, RTX appeared to be both effective and well-tolerated as second or third-line therapy for patients with non-infectious uveitis and scleritis.

## Introduction

While corticosteroids are the first-line therapy for non-infectious uveitis and scleritis, their prolonged use is limited by common side effects [1–3]. Patients requiring long-term immunosuppression for severe disease have been traditionally treated with non-corticosteroid immunosuppressive agents such as methotrexate or azathioprine, leukocyte inhibitors such as cyclosporine or tacrolimus, or with alkylating agents such as cyclophosphamide or chlorambucil [1, 2, 4, 5]. For ocular inflammation refractory to these more traditional therapies, practitioners have increasingly turned to use of biologic agents such as intravenous immunoglobulin, interferons, and tumor necrosis factor (TNF) antagonists [5, 6]. Rituximab (RTX; Rituxan®, Genentech, South San Francisco, CA, USA), a fully humanized anti-CD20 antibody, has gained increasing use and acceptance for the treatment non-infectious ocular inflammation refractory to corticosteroids and traditional immunotherapies. Rituximab causes a depletion of B cells for up to 6 months and is approved by the Food and Drug Administration (FDA) for treatment of non-Hodgkin’s lymphoma, chronic lymphocytic leukemia, rheumatoid arthritis (RA), and granulomatosis with polyangiitis (GPA, formerly Wegener’s Granulomatosis) and microscopic polyangiitis [6–8]. Use of RTX has been described in over 100 cases each of refractory non-infectious uveitis and scleritis, as summarized below.

## Methods

The authors conducted a literature search using the National Library of Medicine’s PubMed database for all English language articles published through December 2020 with the following search terms: “rituximab AND eye”, “rituximab AND uveitis,” and “rituximab AND scleritis.” Use of RTX for orbital inflammation was summarized in a separate companion review [9]. Relevant references within these articles were also reviewed. Included here were all cases of non-infectious scleritis and uveitis for which individual case data was available. Articles describing large series of patients in which individual case data was not provided were not included in the current analysis, but were read for content and referenced when appropriate. Individual information on patient age, sex, anatomical localization and cause of disease, treatment prior to initiation of RTX, time from diagnosis to initiation of RTX, visual acuity before RTX treatment began, visual acuity at last visit, RTX treatment regimen, total number of RTX cycles and interval between cycles, therapeutic response, treatments following initiation of RTX therapy, duration of follow-up and whether disease recurrence occurred, and adverse events attributed to RTX were collected when available. Therapeutic response varied from study to study. In this review, a patient was considered to have had a positive therapeutic response to RTX if they achieved disease quiescence, showed a two-step or more improvement in inflammation as graded by Standardization of Uveitis Nomenclature group [10], showed relative or absolute sparing of corticosteroids or non-corticosteroid medications, or if the authors subjectively documented improvement. Line of therapy was tallied according to the following criteria: first-line – RTX initiated before or at same time as corticosteroids; second-line – RTX initiated after corticosteroids – either alone or with a second more traditional immunosuppressive agent; third-line or greater – RTX initiated after a non-corticosteroid immunosuppressive agent, such as nonbiologic or biologic disease modifying antirheumatic drug, alkylating agents, or intravenous immunoglobulins, with or without corticosteroids. Treatment regimens were classified into either the rheumatologic protocol (two doses of 1000 mg separated by 14 days) [11], the oncologic protocol (four doses of 375 mg/m^2^ weekly) [12], the Foster protocol (eight doses of 375 mg/m^2^ weekly) [13], or other category for the less commonly utilized dosing protocols. Univariate comparisons were made with two-tailed T-test and nomimal, uncorrected *p*-values were reported.

## Results

### Uveitis (Tables 1, 2)

There have been a total of 31 reports describing 108 patients who received treatment with RTX for non-infectious uveitis [14, 15, 16, 17, 18, 19, 20, 21, 22, 23, 24, 25, 26, 27, 28, 29, 30, 31–45]. There was a male to female ratio of 0.26 to 1, and patients possessed a mean age of 39.8 ± 19.4 years (range = 8.0 to 86.0 years; median = 40.0 years). The anatomical location of uveitis was most commonly posterior uveitis (57/108, 52.8%), followed by anterior uveitis (30/108, 27.8%), panuveitis (13/108, 12.0%), unspecified uveitis (4/108, 3.7%), anterior and intermediate uveitis (2/108, 1.9%), intermediate uveitis (1/108, 0.9%), and anterior and posterior uveitis (1/108, 0.9%). Underlying systemic conditions included non-paraneoplastic autoimmune retinopathy (npAIR; 30/107, 28.0%), juvenile idiopathic arthritis (JIA; 21/107, 19.6%), Vogt-Koyanagi-Harada (VKH) disease (12/107, 11.2%), Behçet disease (11/107, 10.3%), cancer-associated retinopathy (CAR; 10/107, 9.3%), sarcoidosis (4/107, 3.7%), GPA (3/107, 2.8%), systemic lupus erythematosus (SLE; 2/107, 1.9%), indeterminate etiology (9/107, 8.4%), and one case each (1/107, 0.9%) of melanoma-associated retinopathy (MAR), type 2 essential cryoglobulinemia, birdshot chorioretinopathy (BSCR), Human Leukocyte Antigen (HLA)-B27-associated uveitis, and multiple sclerosis. Among the 57 cases (47.1%) that reported pre-RTX vision, 38.6% (22/57) had vision worse than 20/200, whereas 31.6% (18/57) had vision better than or equal to 20/40, and 29.8% (17/57) had vision between 20/40 and 20/200.

**Table 1:** Rituximab use in refractory non-infectious uveitis and scleritis - summary of comprehensive literature review

	Non-Infectious Uveitis	Non-Infectious Scleritis
Number of Studies	31	36
Total Patients	108	121
Age (years)	Mean 39.8±19.4 Median 40.0 Range 8.0-86.0	Mean 48.5±16.6 Median 52.0 Range 16.0-81.0
Gender	0.26:1 (M:F)	0.56:1 (M:F)
Ocular Condition Treated with Rituximab (Ratio, %)	Posterior uveitis (57/108, 52.8%); Anterior uveitis (30/108, 27.8%); Panuveitis (13/108, 12.0%); Unspecified uveitis (4/108, 3.7%); Anterior and intermediate uveitis (2/108, 1.9%); Intermediate uveitis (1/108, 0.9%); Anterior and posterior uveitis (1/108, 0.9%);	Anterior scleritis (71/121, 58.7%); Episcleritis (7/121, 5.7%); Posterior scleritis (7/121, 5.7%); Anterior and posterior scleritis (3/121, 2.5%); Location unspecified (33/121, 27.3%)
Underlying Systemic Condition (Ratio, %)	npAIR (30/107, 28.0%) [14–22]; Juvenile idiopathic arthritis (21/107, 19.6%) [23–26]; Vogt-Koyanagi-Harada disease (12/107, 11.2%) [27–30]; Behçet disease (11/107, 10.3%) [31, 32]; Cancer-associated retinopathy (10/107, 9.3%) [14, 33–36]; Sarcoidosis (4/107, 3.7%) [37]; Granulomatosis with polyangiitis (3/107, 2.8%) [38–40]; Systemic lupus erythematosus (2/107, 1.9%) [40]; Birdshot chorioretinopathy (1/107, 0.9%) [41]; HLA-B27 (1/107, 0.9%) [40]; Multiple sclerosis (1/107, 0.9%) [42]; Melanoma-associated retinopathy (1/107, 0.9%) [14]; Type 2 essential cryoglobulinemia (1/107, 0.9%) [43]; Indeterminate (9/107, 8.4%) [24, 40, 44, 45]	GPA (75/121, 62.0%) [11, 24, 38, 39, 46–66]; Rheumatoid arthritis (15/121, 12.4%) [48, 55, 60, 67–70]; ANCA-associated vasculitis, NOS (5/121, 4.1%) [37]; GPA and Rheumatoid arthritis (3/121, 2.5%) [56]; GPA and IgG4-related disease (1/121, 0.8%) [71]; Microscopic polyangiitis (1/121, 0.8%) [60]; Sjogren's syndrome (1/121, 0.8%) [12]; HLA-B27 (1/121, 0.8%) [40]; Behcet's disease (1/121, 0.8%) [24]; Mixed connective tissue disease and Scleroderma (1/121, 0.8%) [55]; IgG4-related disease (1/121, 0.8%) [72]; Cogan syndrome (1/121, 0.8%) [48]; Indeterminate (15/121, 12.4%) [48, 55, 60, 70, 73, 74]
Treatment Prior to Rituximab (Ratio, %)	Corticosteroid only (18/90, 20.0%) 1 Steroid sparing agent (19/90, 21.1%); 2 Steroid sparing agents (11/90, 12.2%); ≥3 Steroid sparing agents (37/90, 41.1%); None (5/90, 5.6%)	Corticosteroid only (4/94, 4.3%) 1 Steroid sparing agent (30/94, 31.9%) 2 Steroid sparing agents (27/94, 28.7%) ≥3 Steroid sparing agents (31/94, 33.0%); None: (2/94, 2.1%)
Previously Utilized Immunosuppressants (Ratio, %)	Corticosteroids (69/85, 81.2%); methotrexate (39/85, 45.9%); cyclosporine (30/85, 35.3%); adalimumab (24/85, 28.2%); infliximab (24/85, 28.2%); mycophenolate mofetil (24/85, 28.2%); etanercept (15/85, 17.6%); azathioprine (11/85, 12.9%); IVIG (8/85, 9.4%); chlorambucil (4/85, 4.7%); leflunomide (4/85, 4.7%); cyclophosphamide (3/85, 3.5%); hydroxychloroquine (3/85, 3.5%) tacrolimus (2/85, 2.4%); abatacept (2/85, 2.4%) anakinra (1/85, 1.2%) sulfasalazine (1/85, 1.2%); interferon-alpha (1/85, 1.2%); bortezomib (1/85, 1.2%)	Corticosteroids (62/90, 68.9%); cyclophosphamide (55/90, 61.1%); methotrexate (42/90, 46.7%); mycophenolate mofetil (24/90, 26.7%); azathioprine (24/90, 26.7%); infliximab (12/90, 13.3%); NSAID (11/90, 12.2%); etanercept (10/90, 11.1%); adalimumab (9/90, 10.0%); cyclosporine (8/90, 8.9%); leflunomide (6/90, 6.7%); hydroxychloroquine (3/90, 3.3%); IVIG (2/90, 2.2%); sulfasalazine (2/90, 2.2%); doxycycline (2/90, 2.2%); Unspecified anti-TNF agent (2/90, 2.2%); anakinra (1/90, 1.1%); minocycline (1/90, 1.1%); interferon alpha (1/90, 1.1%); golimumab (1/90, 1.1%); Abatacept (1/90, 1.1%); Chlorambucil (1/90, 1.1%); Tocilizumab (1/90, 1.1%); Cytarabine (1/90, 1.1%)
Line of Therapy (Ratio, %)	First line (5/90, 5.6%); Second line (18/90, 20.0%); Third or greater (67/90, 74.4%)	First line: (2/96, 2.1%); Second line: (4/96, 4.2%); Third line: (90/96, 93.8%)
Treatment Regimen and Number of Cycles (Ratio, %)	Rheumatologic protocol (45/87, 51.7%); Other (19/87, 21.8%); Foster protocol (18/87, 20.7%); Oncologic protocol (5/87, 5.7%). 1 treatment cycle: (27/88, 30.7%); 2 treatment cycles: (26/88, 29.5%) >2 treatment cycles: (35/88, 39.8%)	Rheumatologic protocol (87/114, 76.3%); Other (11/114, 9.6%); Foster protocol (10/114, 8.8%); Oncologic protocol (6/114, 5.3%) 1 treatment cycle: (34/95, 35.8%); 2 treatment cycles: (18/95, 18.9%); >2 treatment cycles: (43/95, 45.3%)
Types of Responses (Ratio, %)	Responsive (81/97, 83.5%) - Disease Remission (51/81, 63.0%) - Author report: (30/81, 37.0%) Nonresponsive (16/97, 16.5%)	Responsive (112/120, 93.3%) - Disease Remission: (99/112, 88.4%) - Author report: (13/112, 11.6%) Non-responsive (8/120, 6.7%)
Incidence of Recurrence (Ratio, %)	24/81, 29.6%	33/107, 30.8%
Adverse Events (Ratio, %)	None (74/97, 76.3%); Unspecified Infusion reaction (6/97, 6.2%); neutropenia (2/97, 2.1%); conjunctivitis (2/97, 2.1%); GI discomfort (1/97, 2.1%); herpes zoster (1/97, 2.1%); leukopenia (1/97, 1.0%); pneumonia (1/97, 1.0%); flushing (1/97, 1.0%); ventricular tachycardia (1/97, 1.0%); Staphylococcus aureus skin infection (1/97, 1.0%); fungal skin infection (1/97, 1.0%); unspecified dermatitis (1/97, 1.0%); UTI (1/97, 1.0%); Sinusitis (1/97, 1.0%); Abdominal pain, dryness, eyelid swelling (1/97, 1.0%); Sinusitis, herpes zoster, nodular scleritis (1/97, 1.0%)	None (71/83, 85.5%); Itching (infusion reaction) (2/83, 2.4%); Disease exacerbation (2/83, 2.4%); Pneumonia and septic shock (1/83, 1.2%); Herpetic mouth ulcers (1/83, 1.2%); Acute retinal necrosis (1/83, 1.2%); Herpes zoster (1/83, 1.2%); Hypotension (Infusion reaction) (1/83, 1.2%); Ocular hypertension (1/83, 1.2%); Dry eyes, weight loss, fatigue, and joint pain (1/83, 1.2%); Bronchitis, bacterial pneumonia, leukopenia, and anemia (1/83, 1.2%)

*RTX* rituximab, *M* male, *F* female, *npAIR* non-paraneoplastic autoimmune retinopathy, *GPA* granulomatosis with polyangiitis, *NOS* not otherwise specified, *HLA* human leukocyte antigen, *ANCA* Anti-Neutrophilic Cytoplasmic Autoantibody, *NOS* not otherwise specified, *Rheumatologic* Two doses of 1000 mg separated by 14 days, *Oncologic* four doses of 375 mg/m2 weekly, *Foster* eight doses of 375 mg/m2 weekly, *Other* all other RTX dosing regimens, *NSAID* nonsteroidal anti-inflammatory drug, *IVIG* intravenous immunoglobulin, *TNF* tumor necrosis factor, *First line* RTX initiated before or as same time as corticosteroids, *Second line* RTX initiated after corticosteroids, *Third line* RTX initiated after corticosteroids and another agent, such as nonbiologic or biologic disease modifying antirheumatic drug, anti-cancer medications, or intravenous immunoglobulins

**Table 2:** Clinical characteristics of non-infectious Uveitis cases treated with Rituximab

Identified cause of Uveitis	Number (Percentage)	Age (years)	Gender (M:F)	Anatomical Localization (Ratio, %)	Time from Diagnosis to Rituximab Treatment (months)	Rituximab Regimen (Ratio, %)	Positive Therapeutic Response (Ratio, %)	Recurrence (Ratio, %)	Adverse Events (Ratio, %)
Non-paraneoplastic autoimmune retinopathy	30 (28.0%)	Mean: 50.7 ± 16.7 Median: 52.5 Range: 10.0–75.0	0.25:1	Posterior uveitis (30/30, 100%)	Mean: 18.4 Median: 15.0 Range: 2.0–48.0	Rheumatologic (7/30, 23.3%); Oncologic (3/30, 10.0%); Foster (6/30, 20.0%); Other (5/30, 16.7%); Rheumatologic or oncologic (9/30, 30.0%)	24/30, 80.0%	1/24, 4.2%	None (17/26, 65.4%); Unspecified infusion reaction (4/26, 15.4%); Sinusitis (1/26, 7.7%); Infusion reaction with tachycardia (1/26, 3.8%); Leukopenia (1/26, 3.8%); unspecified dermatitis (1/26, 3.8%); Sinusitis, Herpes zoster, nodular scleritis (1/26, 3.8%)
Juvenile idiopathic arthritis	21 (19.6%)	Mean: 20.7 ± 5.7 Median: 21.0 Range: 13.0–34.0	0.12:1	Anterior uveitis (18/21, 85.7%); Unspecified uveitis (2/21, 9.5%); Anterior and intermediate uveitis (1/21, 4.8%);	Mean: 48.0Median: 36.0 Range: 24.0–168.0	Rheumatologic (11/21, 52.4%); Two infusions of 375 mg/m^2^ separated by 2 weeks (10/21, 47.6%)	16/21, 76.2%	11/16, 68.8%	None (21/21, 100%)
Vogt-Koyanagi-Harada disease	12 (11.2%)	Mean: 23.4 ± 10.8 Median: 21.0 Range: 8.0–41.0	100% F	Anterior uveitis (8/11, 72.7%); Panuveitis (3/11, 27.3%)	Mean: 49.5 Median: 36.0 Range: 10.0–156.0	Rheumatologic (8/12, 66.7%); Other (4/12, 33.3%)	12/12, 100%	0/12, 0%	None (10/10, 100%)
Behcet’s disease	11 (10.3%)	Mean: 28.8 ± 10.7 Median: 28.0 Range: 17.0–51.0	1.75:1	Posterior uveitis (11/11, 100%)	NR	Rheumatologic (11/11, 100%)	11/11, 100%	10/11, 90.9%^b^	None (6/11, 54.5%); Conjunctivitis (2/11, 18.2%); Pneumonia (1/11, 9.1%); Herpes zoster (1/11, 9.1%); Flushing (1/11, 9.1%); Mild urticaria (1/11, 9.1%)
Cancer-associated and Melanoma-associated retinopathy	11 (10.3%)^a^	Mean: 63.3 ± 13.5 Median: 61.0 Range: 46.0–86.0	0.38:1	Posterior Uveitis (11/11, 100%)	Mean: 19.1 Median: 12.5 Range: 0–75.0	Either rheumatologic or oncologic protocol (9/11, 81.8%); Unspecified (2/11, 18.2%)	6/11, 54.5%	N/A	None (6/8, 75%); Infusion reaction (2/8, 25%)
Sarcoidosis	4 (3.7%)	Mean: 47.75 Median: 47.5 Range: 46.0–50.0	100% F	Panuveitis (3/4, 75.0%) Unspecified uveitis (1/4, 25.0%)	NR	Rheumatologic (4/4, 100%)	3/4, 75.0%	NR	Neutropenia (2/4, 50.0%); Staphylococcus aureus skin infections (1/4, 25.0%); None (1/4, 25.0%)
Other^c^	9 (8.4%)	Mean: 48.0 ± 9.6 Median: 42.0 Range: 37.0–62.0	0.5:1	Posterior uveitis (3/9, 33.3%); Panuveitis (2/9, 22.2%); Anterior uveitis (1/9, 11.1%); Anterior and intermediate uveitis (1/9, 11.1%); Anterior uveitis and vasculitis (1/9, 11.1%); Unspecified uveitis and vasculitis (1/9, 11.1%)	NR	Rheumatologic (3/8, 37.5%); Foster (4/8, 50.0%); Other (1/8, 12.5%)	5/5, 100%	1/5, 20.0%	None (3/3, 100%)
Indeterminate	9 (8.4%)	Mean: 43.2 ± 19.0 Median: 49.0 Range: 14.0–65.0	0.13:1	Panuveitis (5/9, 55.6%); Posterior uveitis (2/9, 22.2%); Anterior uveitis (2/9, 22.2%)	NR	Foster(7/9, 77.8%); Rheumatologic (1/9, 11.1%); Oncologic (1/9, 11.1%)	4/4, 100%	1/4, 25%	None (1/3, 33.3%); Fungal skin infection (1/3, 33.3%); Gastrointestinal discomfort (1/3, 33.3%)

*M* male, *F* female, *NR* not reported, Rheumatologic = Two doses of 1000 mg separated by 14 days, Oncologic = four doses of 375 mg/m2 weekly, Foster = eight doses of 375 mg/m2 weekly, Other = all other RTX dosing regimens

^a^ = 10 cases of cancer-assocaited retinopathy, and one case of melanoma-associated retinopathy

^b^ = all patients only received one cycle of rituximab, recurrences occurred between 6 and 13 months, with a median of 7.5 months, after treatment

^c^ = includes 3 cases of GPA, 2 cases of SLE, and 1 case each of HLA-B27, multiple sclerosis, type 2 essential cryoglobulinemia, and birdshot chorioretinitis

In the 90 cases with documented treatment history, a total of 74.4% (67/90) of patients received RTX as third-line or later therapy, followed by 20.0% (18/90) as second-line, and 5.6% (5/90) as first-line. Previous therapies included none (5/90, 5.6%), corticosteroids alone (18/90, 20%), or one (19/90, 21.1%), two (11/90, 12.2%), or three or more (37/90, 41.1%) corticosteroid-sparing immunosuppressive agents with or without corticosteroids. Immunosuppressive agents tried prior to RTX included corticosteroids (69/85, 81.2%), methotrexate (39/85, 45.9%), cyclosporine (30/85, 35.3%), adalimumab (24/85, 28.2%), infliximab (24/85, 28.2%), mycophenolate mofetil (24/85, 28.2%), etanercept (15/85, 17.6%), azathioprine (11/85, 12.9%), intravenous immunoglobulins (8/85, 9.4%), chlorambucil (4/85, 4.7%), leflunomide (4/85, 4.7%), hydroxychloroquine (3/85, 3.5%), cyclophosphamide (3/85, 3.5%), tacrolimus (2/85, 2.4%), abatacept (2/85, 2.4%), anakinra (1/85, 1.2%), sulfasalazine (1/85, 1.2%), interferon-alpha (1/85, 1.2%), and bortezomib (1/85, 1.2%). The mean time from diagnosis to RTX use was 33.5 ± 34.7 months (range = 0 to 168.0 months; median = 24.0 months).

Various RTX treatment regimens were utilized to treat patients with non-infectious uveitis, with 51.7% (45/87) receiving the rheumatologic protocol, 21.8% (19/87) receiving a variety of uncommon off-label protocols, 20.7% (18/87) receiving the Foster protocol, and 5.7% (5/87) receiving the oncologic protocol. A total of 30.7% (27/88) patients received just 1 cycle of RTX, leading to disease remission for 81.5% (22/27), no observed response for 18.5% (5/27), and eventual disease recurrence in 54.5% of responders (12/22) at a mean of 8.0 months (range = 6.0 to 13.0 months; median = 7.0 months) despite continued treatment with other forms of systemic immunomodulatory therapies. In total, 69.3% (61/88) of treated patients received between two to 12 cycles of RTX at varying intervals: 4 weeks (20/58, 34.5%) [16, 17, 37, 38, 40], 8 weeks (3/58, 5.2%) [17, 37], 3 to 6 months (1/58, 1.7%) [38], 6 months (25/58, 43.1%) [17, 20, 22, 23, 29, 30] 8 months (1/58 1.7%) [25], 9 months (1/58, 1.7%) [25], 6 to 10 months (1/58, 1.7%) [24], or ≥ 12 months (4/58, 6.9%) [15, 17, 25]. Two studies employed increasing retreatment durations (2/58, 3.4%) [27, 28]. Three studies utilized the Foster protocol, followed by four monthly infusions at the same dose, and then further monthly treatments as needed based on clinical examination, but did not provide individual data on total number of treatments and indication for each retreatment [16, 38, 40]. For the patients with available longitudinal data (47/61, 77.0%), 23.4% (11/47) received two or more cycles of RTX for disease recurrence [23–25, 38]. Otherwise, the rationale of designated treatment intervals and indications for retreatment were unclear in the other reviewed studies.

In reports with available individual data (*n* = 97), 83.5% (81/97) of patients treated with RTX experienced a positive therapeutic response, with 63.0% (51/81) having disease remission and 37.0% (30/81) showing disease improvement or stability based on author report. In contrast, 16.5% (16/97) of patients were described as having treatment failure with RTX. One or more disease recurrences eventually developed in 29.6% (24/81) of the patients responsive to RTX, with mean interval to first disease recurrence of 8.5 months (range = 3 to 17 months; median = 8.0 months). Information on individualized treatment regimens following uncomplicated RTX therapy was available for 78.9% (45/57) of patients who experienced disease remission without recurrence, with 44.4% (20/45) receiving ongoing treatment with one or more non-RTX, corticosteroid-sparing systemic immunosuppressive agents at mean follow up of 17.6 months (range = 1.0 to 36.0 months; median = 18.0 months), 29.3% (12/45) sustaining RTX treatments at mean follow up of 17.1 months (range = 7.0 to 60.0 months; median = 17.1 months), 17.8% (8/45) achieving drug-free remission at mean follow up of 32.6 months (range = 4 to 81 months; median = 22.0 months), and 11.1% (5/45) maintaining disease remission with only topical corticosteroid therapy at a mean follow up 14.2 months (range = 11 to 26 months; median = 11.0 months). Among the patients with reported visual acuities following RTX therapy, 39.2% (20/51) had vision between 20/40 and 20/200, 35.3% (18/51) better than or equal to 20/40, and 25.5% (13/51) were worse than or equal to 20/200.

A total of 76.3% (74/97) of patients who received RTX treatments for non-infectious uveitis reported no adverse events. However, six cases (6/97, 6.2%) of unspecified infusion reactions [14, 20], two cases each (2/97, 2.1%) of neutropenia [37] and conjunctivitis [31], and one case each (1/97, 1.0%) of gastrointestinal discomfort [45], herpes zoster [31], sinusitis [14], leukopenia [14], pneumonia [31], flushing during infusion [31], ventricular tachycardia during infusion [14], *Staphylococcus aureus* skin infection [37], fungal skin infection [44], unspecified dermatitis [16], and urinary tract infection [40] were reported following RTX treatments. Two individuals reported multiple adverse events attributed to RTX; one (1/97, 1.0%) developed abdominal pain, dryness, and eyelid swelling, and the other (1/97, 1.0%) sinusitis, herpes zoster, and nodular scleritis [17, 40].

### Non-paraneoplastic autoimmune retinopathy (npAIR)

Patients with npAIR accounted for 28.0% (30/107) of patients with non-infectious uveitis who received treatment with RTX. There was a 0.25 to 1 male to female ratio, and mean age at time of treatment was 50.7 years (range = 10.0 to 75.0 years; median = 52.5 years). The mean interval from diagnosis of npAIR to initiation of RTX treatment was 18.4 months (range = 2.0 to 48.0 months; median = 15.0 months). One study with nine patients (9/30, 30.0%) utilized either the rheumatologic or oncologic protocols, but did not specify [14]. Otherwise, 23.3% (7/30) of cases were treated according to the rheumatologic protocol, 20% (6/30) the Foster protocol, 16.7% (5/30) with various uncommon off-label treatment regimens, and 10.0% (3/30) the oncologic protocol. A total of 80.0% (24/30) of RTX-treated patients were described as having disease remission or regression following RTX treatment due to improvement or stability in vision, visual fields, and/or electroretinography (ERG). One treatment responder (1/24, 4.2%) developed later disease relapse. Mild infusion reactions (4/26, 15.4%) [14, 20] were the most common reported adverse event, but sinusitis [14], tachycardia following infusion [14], leukopenia [14], unspecified dermatitis [16], and the combination of sinusitis, herpes zoster, and nodular scleritis [17] were reported in one patient each (1/26, 3.8%).

### Cancer-associated retinopathy (CAR) and melanoma-associated retinopathy (MAR)

In total, 10.3% of patients (11/107; 10 CAR, 1 MAR) with non-infectious uveitis were treated with RTX for paraneoplastic retinopathy. These patients had a male to female ratio of 0.38 to 1 and mean age of 63.3 years (range = 46.0 to 86.0 years; median 61.0 years) when RTX treatment began. The mean interval from diagnosis of CAR or MAR to initiation of RTX treatment was 19.1 months (range = 0 to 75.0 months; median = 12.5 months). A total of 81.8% (9/11) of patients received treatment with either the rheumatologic or oncologic protocol, while the dose and frequency was unspecified in 18.2% (2/11). Following RTX treatment, 54.5% (6/11) of cases were assessed to have a positive therapeutic response based on improvement in vision, visual fields, and/or ERG. Overall, 75.0% (6/8) reported no adverse events following RTX treatments, but unspecified infusion reactions were reported in two patients (2/8, 25.0%) [14].

### Juvenile idiopathic arthritis

A total of 19.6% (21/107) of patients who received RTX for non-infectious uveitis possessed a systemic diagnosis of JIA. These patients possessed a male to female ratio of 0.12 to 1 and mean age of 20.7 years (range = 13.0 to 34.0 years, median = 21.0 years) at time of treatment initiation. The mean interval from diagnosis of uveitis to treatment with RTX was 48.0 months (range = 24.0 to 168.0 months; median = 36.0 months). Overall, 85.7% (18/21) possessed anterior uveitis, 9.5% (2/21) unspecified uveitis, and 4.8% (1/21) anterior and intermediate uveitis. In total, 52.4% (11/21) of patients received treatment with the rheumatologic protocol, and 47.6% (10/21) with a modified rheumatologic protocol using 375 mg/m^2^ RTX dosing instead of 1 g. Following treatment with RTX, 76.2% (16/21) of recipients experienced a positive therapeutic response, but 68.8% (11/16) of responders eventually developed disease recurrence. No adverse events were reported in this subgroup.

### Vogt-Koyanagi-Harada (VKH) disease

Patients with VKH disease accounted for 11.2% (12/107) of patients with non-infectious uveitis who were treated RTX. All patients were female and possessed a mean age of 23.4 years (range = 8.0 to 41.0 years; median = 21.0 years) at time of RTX therapy. The mean interval from initial diagnoses of uveitis to RTX treatment was 49.5 months (range = 10.0 to 156.0 months; median = 36.0 months). In total, 72.7% (8/11) of patients were diagnosed with anterior uveitis and 27.3% (3/11) with panuveitis. A total of 66.7% (8/12) were treated according to the rheumatologic protocol, and the remaining 33.3% (4/12) with a variety of uncommon off-label regimens. All subjects received two or more doses without any reported adverse events and were described to achieve disease remission without relapse at mean follow up of 19.8 months (range = 9.0 to 36.0 months; median = 17.0 months).

### Behçet disease

In total, 10.3% (11/107) of patients who received RTX for non-infectious uveitis possessed various forms of posterior uveitis caused by Behçet disease. Gender distribution was 63.6% (7/11) male and 36.4% (4/11) female, and mean age at time of treatment was 28.8 years (range = 17.0 to 51.0 years; median = 28.0 years). All patients were treated with just 1 cycle of the rheumatologic protocol (11/11, 100%) and were reported to have a positive therapeutic response (11/11, 100%) with disease remission, but there was a 90.9% (10/11) rate of subsequent disease relapse occurring at mean interval of 8.2 months (range = 6.0 to 13.0 months; median = 7.5 months). Overall, 54.5% (6/11) of treated patients reported no adverse events, but two patients each (2/11, 18.2%) developed conjunctivitis and mild infusion reactions, and one patient each (1/11, 9.1%) developed unspecified pneumonia and herpes zoster attributed to RTX [31].

### Uveitis of indeterminate etiology

A total of 8.4% (9/107) of patients with non-infectious uveitis who received RTX treatment possessed no discernable systemic etiology for their ocular inflammation. Gender distribution was 88.9% (8/9) female and 11.1% (1/9) male, and the mean age at time of RTX therapy was 43.2 years (range = 14.0 to 65.0 years; median = 49.0 years). In total, 55.6% (5/9) of patients possessed panuveitis and 22.2% (2/9) each possessed posterior uveitis and anterior uveitis. Overall, 77.8% (7/9) of cases were treated according to the Foster protocol, and 11.1% (1/9) each the rheumatologic and oncologic protocols. Individualized longitudinal patient data was limited but showed a 100.0% (4/4) rate of positive therapeutic response, with a 25.0% (1/4) rate of eventual disease recurrence. Information regarding tolerability of RTX was available for three patients, with one report each of (1/3, 33.3%) gastrointestinal discomfort and fungal cellulitis attributed to RTX [44, 45].

### Scleritis (Tables 1, 3)

There have been a total of 36 reports describing 121 patients who received treatment with RTX for non-infectious scleritis [11, 12, 24, 37–40, 46–65, 66, 67–70, 71, 72, 73, 74]. These patients possessed a male to female ratio of 0.56 to 1 and a mean age of 48.5 ± 16.6 years (range = 16.0 to 81.0 years; median = 52.0 years) when first treated with RTX. The cases of refractory non-infectious scleritis were attributed to a wide range of underlying systemic conditions including GPA (75/121, 62.0%), RA (15/121, 12.4%), indeterminate etiology (15/121, 12.4%), antineutrophil cytoplasmic antibody (ANCA)-associated vasculitis without further specification (5/121, 4.1%), both GPA and RA (3/121, 2.5%), and one case each (1/121, 0.8%) of GPA and Immunoglobulin G4 (IgG4)-related disease, IgG4-related disease alone, Behçet disease, Cogan syndrome, primary Sjogren’s syndrome, mixed connective tissue disease and scleroderma, microscopic polyangiitis, and HLA-B27 associated disease. Visual acuity prior to RTX therapy was reported in only 29 cases, with 62.1% (18/29) of patients having vision better than or equal to 20/40, 24.1% (7/29) worse than or equal to 20/200, and 13.8% (4/29) with vision between 20/40 and 20/200.

**Table 3:** Clinical Characteristics of Non-infectious Scleritis Cases Treated with Rituximab

Identified cause of Scleritis	Number (Percentage)	Age (years)	Gender	Anatomical Localization (Ratio, %)	Time from Diagnosis to Rituximab Treatment (months)	Rituximab Regimen (Ratio, %)	Positive Therapeutic Response (Ratio, %)	Recurrence (Ratio, %)	Adverse Events (Ratio, %)
Granulomatosis with Polyangiitis (GPA)	75 (62.0%)	Mean: 48.2 ± 16.0 Median: 50.0 Range: 21.0–72.0	M:F 0.80:1	Unspecified scleritis (35/75, 46.7%); Anterior scleritis (34/75, 45.3%); Posterior scleritis (3/75, 4.0%); Episcleritis (2/75, 2.7%); Anterior and posterior scleritis (1/75, 1.3%)	Mean: 30.9 ± 29.5 Median: 21 Range: 1–96	Rheumatologic (57/68, 83.8%); Foster (4/68, 5.9%); Other (4/68, 5.9%); Oncologic (3/68, 4.4%)	71/73, 97.3%	21/73, 28.8%	None (40/45, 88.9%); Disease exacerbation (2/45, 4.4%); Herpes zoster (1/45, 2.2%); Ocular hypertension (1/45, 2.2%); Dry eyes, weight loss, fatigue, joint pain (1/45, 2.2%)
Rheumatoid arthritis (RA)	15 (12.4%)	Mean: 53.3 ± 12.2 Median: 55.0 Range: 27.0–74.0	M:F 1:0.83	Anterior scleritis (8/15, 53.3%); Unspecified scleritis (6/15, 40.0%); Anterior and posterior scleritis (1/15, 6.7%)	NR	Rheumatologic (10/11, 90.9%); Foster (1/11, 9.1%)	11/11, 100%	2/10, 20.0%	None (8/11, 72.7%); Infusion hypotension (1/11, 9.1%); Acute retinal necrosis (1/11, 9.1%); Itching with infusion (1/11, 9.1%)
GPA and RA	3 (2.5%)	NR	M:F 0.5:1	Anterior scleritis (2/3, 66.7%) Anterior and posterior scleritis (1/3, 33.3%)	Mean: 9 Median: 8 Range: 5–14	Rheumatologic (2/3, 66.7%); Oncologic (1/3, 33.3%)	3/3, 100%	2/3, 66.7%	None (2/3, 66.7%); Bronchitis (1/3, 33.3%); Bacterial pneumonia (1/3, 33.3%); Leukopenia (1/3, 33.3%); Anemia (1/3, 33.3%)
ANCA-associated vasculitis, not otherwise specified	5 (4.1%)	Mean: 59.6 Median: 59.0 Range: 41.0–78.0	M:F0.67:1	Episcleritis (5/5, 100%)	NR	Rheumatologic (5/5, 100%)	5/5, 100%	NR	None (4/4, 100%)
Other^a^	8 (6.7%)	Mean: 52.0 ± 23.7 Median: 53.0 Range: 17.0–81.0	M:F 0.4:1	Anterior scleritis (4/8, 50.0%); Unspecified scleritis (3/8, 37.5%); Posterior scleritis (1/8, 12.5%)	NR	Rheumatologic (3/7, 42.9%); Foster: (2/7, 28.6%); Other: (2/7, 28.6%)	6/7, 85.7%	0/6, 0%	None (3/4, 75.0%); Pneumonia and septic shock (1/4, 25.0%)
Indeterminate	15 (12.4%)	Mean: 37.4 ± 15.0 Median: 43.0 Range: 16.0–57.0	M:F 0.22:1	Anterior scleritis (9/15, 60.0%); Posterior scleritis (2/15, 13.3%); Unspecified scleritis (4/15, 26.7%)	NR	Rheumatologic (3/8, 37.5%); Oncologic (2/8, 25.0%); Foster (2/8, 25.0%); Other (1/8, 12.5%)	7/9, 77.8%	1/7, 14.3%	None (14/15, 93.3%); Itcing with infusion (1/15, 6.7%)

*M* male, *F* female, *NR* not reported, Rheumatologic = Two doses of 1000 mg separated by 14 days, Oncologic = four doses of 375 mg/m2 weekly, Foster = eight doses of 375 mg/m2 weekly, Other = all other RTX dosing regimens

^a^ = one case each of microscopic polyangiitis, Sjogren’s syndrome, HLA-B27, Behcet’s disease, mixed connective tissue disease/scleroderma, IgG4-related disease, IgG4-related disease and granulomatosis with polyangiitis, and Cogan syndrome

In the 96 cases with documented treatment history, a total of 93.8% (90/96) of patients received RTX as third-line or later therapy, followed by 4.2% (4/96) as second-line, and 2.1% (2/96) as first-line. Treatment regimens prior to RTX were either none (2/94, 21.%), corticosteroids alone (4/94, 4.3%), or one (30/94, 31.9%), two (27/94, 28.7%), or three or more (31/94, 33.0%) corticosteroid-sparing immunosuppressive agents with or without corticosteroids. Previous immunomodulatory therapies included corticosteroids (62/90, 68.9%), cyclophosphamide (55/90, 61.1%), methotrexate (42/90, 46.7%), mycophenolate mofetil (24/90, 26.7%), azathioprine (24/90, 26.7%), infliximab (12/90, 13.3%), nonsteroidal anti-inflammatory drugs (11/90, 12.2%), etanercept (10/90, 11.1%), adalimumab (9/90, 10.0%), cyclosporine (8/90, 8.9%), leflunomide (6/90, 6.7%), hydroxychloroquine (3/90, 3.3%), sulfasalazine (2/90, 2.2%), intravenous immunoglobulin (2/90, 2.2%), doxycycline (2/90, 2.2%), and one case each (1/90, 1.1%) of cytarabine, interferon-alpha, or golimumab, anakinra, minocycline, tocilizumab, and abatacept. The mean interval from diagnosis of scleritis to treatment with rituximab was 39.4 ± 36.6 months (range = 1.0 to 168.0 months; median = 21.0 months).

Various RTX treatment regimens were utilized to treat cases non-infectious scleritis, with 76.3% (87/114) receiving the rheumatologic protocol, 9.6% (11/114) receiving a variety of uncommon off-label protocols, 8.8% (10/114) receiving the Foster protocol, and 5.3% (6/114) receiving the oncologic protocol. A total of 35.8% (34/95) of patients received just 1 cycle of RTX, leading to a positive response with disease remission in 84.3% (29/34), treatment failure in 14.7% (5/34), and disease relapse in 6.9% (2/29). The remaining 64.2% (61/95) of treated patients received between two to 15 cycles of RTX at intervals ranging from 1 to 6 months, with 18.0% (11/61) receiving one retreatment for disease relapse [48, 61, 68, 70]. However, the rationale of designated treatment intervals and indications for retreatment in other reports were unclear. Of note, three patients developed disease recurrence despite receiving RTX treatment cycles every 4 to 6 months [55, 57], and two patients experienced relapse while receiving repeat infusions of unreported interval [47].

Following treatment with RTX for non-infectious scleritis, 93.3% (112/120) of patients experienced a positive therapeutic response, with 88.4% (99/112) experiencing disease remission for at least some period of time, and 11.6% (13/112) showing disease improvement based on author report. In contrast, 6.7% (8/120) of patients were described as having treatment failure with RTX. One or more disease recurrences eventually developed in 30.8% (33/107) of the patients responsive to RTX, with a mean interval to first disease recurrence of 14.4 months (*n* = 9; range = 4.0 to 55.0 months; median = 6.0 months). In a series of 20 patients with GPA-associated scleritis treated with RTX, Joshi et al. reported a 95.0% (19/20) response rate followed by a 63.2% (12/19) rate of relapse occurring at a median of 27.0 months following treatment (mean and range unreported) [46]. In another series of 12 patients with refractory scleritis arising from rheumatoid arthritis, GPA, Cogan syndrome, or an indeterminate etiology, Suhler et al. reported a 75.0% (9/12) response rate following RTX treatment followed by a 77.8% (7/9) rate of eventual disease relapse at a mean of 32.0 weeks (range = 24.0 to 46.0 weeks, median unreported) [48]. Information on individualized treatment regimens following uncomplicated RTX therapy was available for 83.8% (62/74) of patients with disease remission without recurrence, showing that 40.3% (25/62) continued maintenance immunosuppression with one or more non-RTX, corticosteroid-sparing systemic immunosuppressive agents at mean follow up of 18.0 months (range = 2.0 to 48.0 months; median = 12.0 months), 35.5% (22/62) maintained ongoing RTX treatments at mean follow up of 28.9 months (range = 6.0 to 103.0 months; median = 24.0 months), and 24.2% (15/62) achieved drug-free remission at mean follow up of 21.5 months (range = 1.0 to 49.0 months; median = 16.0 months). For the 26 cases that reported visual acuities following RTX treatment, 50.0% (20/40) of patients retained 20/40 or better vision, 26.9% (7/26) vision between 20/40 and 20/200, and 23.1% (6/26) vision equal to or worse than 20/200.

A total of 85.5% (71/83) reported no adverse events following treatment with RTX for non-infectious scleritis. However, two patients each (2/83, 2.4%) developed itching with infusion [70] and disease exacerbation [58, 62], and one patient each (1/83, 1.2%) developed herpetic mouth ulcers [37], acute retinal necrosis [67], herpes zoster [56], hypotension during infusion of RTX [55], ocular hypertension [57], pneumonia with septic shock [60], dry eyes, the combination of weight loss, fatigue, and joint pain [63], and the combination of leukopenia, anemia, bronchitis, and bacterial pneumonia [56] attributed to RTX treatments.

### Granulomatosis with Polyangiitis

In total, 62.0% (75/121) of patients with non-infectious scleritis who received RTX treatment possessed an underlying diagnosis of GPA. These patients had a male to female ratio of 0.80 to 1 and a mean age of 48.2 ± 16.0 years (range = 21.0 to 72.0 years; median = 50.0 years) when treatment began. In total, 46.7% (35/75) of these patients possessed unspecified scleritis, 45.3% (34/75) possessed anterior scleritis, and possessed 4.0% (3/75) posterior scleritis. The mean interval from diagnosis of scleritis to initiation of RTX treatment was 30.9 months (range = 1.0 to 96.0 months; median = 21.0 months). A total of 83.8% (57/68) were treated according to the rheumatologic protocol, 5.9% (4/68) each the Foster protocol and various uncommon off-label regimens, and 4.4% (3/68) the oncologic protocol. A positive therapeutic response was reported for 97.3% (71/73) of treated patients, with a 28.8% (21/73) rate of subsequent disease relapse. In total, 88.9% (40/45) experienced complication free treatment, but two patients (2/45, 4.4%) developed disease exacerbation [58, 62], and one patient each (1/45, 2.2%) developed herpes zoster [56], ocular hypertension [57], and the nonspecific constellation of dry eyes, weight loss, fatigue, and joint pain [63] attributed to RTX treatment.

### Rheumatoid arthritis

A total of 12.4% (15/121) of patients received RTX as treatment for non-infectious scleritis that was attributed to RA. These patients possessed a male to female ratio of 1 to 0.83 and a mean age of 53.3 years (range = 27.0 to 74.0 years; median = 55.0 years) when treatment began*.* In total, 53.3% (8/15) of patients possessed anterior scleritis, 40.0% (6/15) possessed unspecified scleritis, and 6.7% (1/15) possessed both anterior and posterior scleritis. A total of 90.9% (10/11) of cases were treated according to the rheumatologic protocol and 9.1% (1/11) by the Foster protocol. Authors reported 100% (10/10) positive therapeutic response following RTX treatment, with a 20.0% (2/10) rate of eventual disease relapse. Overall, 72.7% (8/11) of patients experienced no adverse events following RTX treatment, but one patient each (1/11, 9.2%) developed hypotension with infusion [55], itching with infusion [70], and acute retinal necrosis [67] attributed to RTX.

### Scleritis of indeterminate etiology

In total, 12.4% (15/121) of patients with non-infectious scleritis treated with RTX possessed no discernable systemic etiology for their ocular inflammation. These patients possessed a male to female ratio of 0.22 to 1 and a mean age of 37.4 years (range = 16.0 to 57.0 years; median = 43.0 years) when treatment began. A total of 60.0% (9/15) of patients were diagnosed with anterior scleritis, followed by 26.7% (4/15) with unspecified scleritis, and 13.3% (2/15) with posterior scleritis. Overall, 37.5% (3/8) of patients were treated according to the rheumatologic protocol, 25.0% (2/8) each according to the Foster and oncologic protocols, and 12.5% (1/8) with an off-label regimens. Following RTX therapy, 77.8% (7/9) of patients experienced a positive therapeutic response, with a 14.3% (1/7) rate of eventual disease recurrence. No adverse events were reported in 93.3% (14/15) of treated individuals, but one patient (1/15, 6.7%) developed itching during RTX fusion [70].

## Discussion

Rituximab, a fully humanized monoclonal anti-CD20 antibody, has increasingly been utilized as a safe and efficacious treatment of refractory, non-infectious uveitis or scleritis arising from npAIR, JIA, VKH disease, Behçet disease, GPA, and RA. Rituximab was used as a third-line or later treatment in nearly three-quarters of patients with non-infectious uveitis who had been unresponsive to corticosteroid or other corticosteroid-sparing immunosuppressants, with treatment generally initiated 2 to 3 years following initial diagnosis. Half of these patients received the rheumatologic protocol, one fifth the Foster protocol, another fifth various off-label dosing regimens, and the remaining tenth the oncologic protocol. Nearly all patients with non-infectious scleritis refractory to corticosteroid or other corticosteroid sparing immunosuppressants received RTX as a third-line or later agent, with treatment generally initiated 2 to 3 years following initial diagnosis. Three quarters of these patients received the rheumatologic protocol, a tenth various uncommon off-label regimens, another tenth the Foster protocol, and the remaining with the oncologic protocol. Patients with uveitis experienced a positive therapeutic response in more than eight out of 10 cases, with less than a quarter reporting generally mild adverse events that were most commonly injection site reactions. Similarly, patients with scleritis reported disease remission in nearly nine out of 10 cases, with slightly more than a seventh of patients reporting generally mild side effects.

The efficacy of RTX for treatment of non-infectious uveitis and scleritis showed variation according to underlying systemic cause. All of the patients with non-infectious uveitis secondary to VKH disease (*n* = 12, 100%), Behçet disease (*n* = 11, 100%), or uveitis of indeterminate cause (*n* = 4, 100%) had a positive therapeutic response. Similarly, nearly all patients with refractory scleritis from GPA (*n* = 73, 97.3%), RA (*n* = 11, 100%), GPA and RA (*n* = 3, 100%), and unspecified ANCA-associated vasculitis (*n* = 5, 100%) exhibited a favorable therapeutic outcome. Moderately less efficacy was observed for patients with uveitis attributed to npAIR (*n* = 30, 80.0%), JIA (*n* = 21, 76.2%), and sarcoidosis (*n* = 4, 75.0%), and those with scleritis from an indeterminate etiology (*n* = 9, 77.8%). Patients with refractory uveitis from CAR showed a response in just over half (54.5%) of patients treated with RTX, mirroring prior reports regarding the difficult nature of these conditions to control even with various modalities of long-term immunosuppression [75]. Treatment with RTX may be more efficacious for cases of non-infectious scleritis than for uveitis (*p* = 0.02, N-1 two-proportion test, two-tailed). However, when patients with CAR were excluded from analysis, this difference decreased dramatically (*p* = 0.13, N-1 two-proportion test, two-tailed).

Maintenance of disease remission in patients with refractory non-infectious uveitis and scleritis appeared to be more likely following multiple cycles of RTX treatment or other forms of ongoing systemic immunosuppression. The majority of patients who maintained disease remission following RTX treatment either required other forms of systemic immunosuppression (uveitis: 44.4%; scleritis: 40.3%) or ongoing RTX infusions (uveitis: 29.3%; scleritis: 35.5%). A minority of patients were able to attain sustained drug free remission (uveitis: 17.8%; scleritis: 24.2%). Following the first cycle of RTX treatment, just under one-third of patients (uveitis: 29.6%; scleritis: 30.8%) developed disease recurrence.

Given that the patients in this review tended to possess either intractable non-infectious uveitis or scleritis despite treatments with corticosteroid and traditional non-corticosteroid immunosuppressive agents, it was unsurprising that RTX was generally used as a third-line or later medication. Other possible contributing factors could have been the cost of RTX, a relatively more difficult route of administering RTX, and/or the unfamiliarity to providers with RTX as a treatment choice in such patients. From 2005 to 2020, authors consistently used RTX as a third-line (90/95, 94.7%) medication to treat scleritis. While RTX was also used as predominantly a third-line medication (67/90, 74.4%) for treatment of refractory uveitis, some authors began to utilize RTX as a second-line (18/90, 20.0%) agent to treat VKH disease, npAIR, and CAR beginning in 2017 [14, 29]. Of note, RTX was used as a first line-drug for three patients with npAIR and two with CAR [14, 17, 20, 35, 36]. Three of these patients were each part of a larger series wherein other patients received RTX as a third-line agent, and no explanation was provided as to why these patients differed in their treatment approach [14, 17, 20]. Sen et al. and Or et al. both initiated early treatment with RTX in hopes of targeting the B lymphocytes responsible for production of serum antibodies directed against a retinal antigen in the photoreceptor layer [35, 36]. The generally positive outcomes noted here suggest, however, that RTX might be considered earlier in the course of therapy in some patients.

Overall, continued long-term immunosuppression appeared to be more important than selecting a particular treatment protocol given the comparable efficacy of the various treatment regimens, and the proclivity for disease relapse in RTX-treated patients with non-infectious uveitis and scleritis. The rheumatologic protocol (132/201, 65.7%) was by far the most commonly utilized treatment regimen. Other established treatment regimens such as the Foster and oncologic protocols were utilized to treat a minority of patients. Roughly one in six subjects received various other off-label dosing regimens. Following RTX treatment according to the rheumatologic regimen, more than 90% of those with both uveitis and scleritis experienced a positive therapeutic response. Given limited sample sizes and lack of standardization across the studies, the collected data was insufficient to allow for comparison of treatment efficacy between the various regimens. For those cases with individualized dosing data, about one-third were treated with only 1 cycle of RTX leading to disease remission in more than eight out of 10 – although roughly one-quarter of those with initial positive therapeutic response eventually relapsed despite subsequent treatment with other forms of systemic immunomodulatory therapy. Nearly one quarter of cases reported using rituximab for 2 cycles, with half receiving a second cycle in response to disease recurrence. The remaining patients received more than 2 cycles of RTX not for specifically for disease relapse, but also for further disease control, scheduled re-treatments to maintain disease remission, or reasons not otherwise specified.

Clinical trials investigating RTX treatment for rheumatologic diseases have found it to be relatively well-tolerated with mild to moderate infusion-related reactions as the most common adverse response [76–78]. Severe adverse reactions are uncommon, but can include tumor lysis syndrome, severe mucocutaneous reactions, progressive multifocal leukoencephalopathy, hepatitis B reactivation, infections, cardiac arrhythmias, renal toxicity, and bowel obstruction and perforation [79]. Roughly one-fifth (35/180, 19.4%) of RTX-treated patients with non-infectious uveitis and scleritis were reported to have an adverse event. According to the common terminology criteria for adverse events, 40.0% (14/35) were Grade 1 reactions, 37.1% (13/35) were Grade 2 reactions, and 22.8% (8/35) were Grade 3 or 4 reactions; no deaths (Grade 5) were reported. The most common adverse event was an infusion reaction (11/35, 31.4%). Overall, the rate of reported adverse events did not differ significantly from rates reported in the literature [76–78].

Our retrospective analysis had limitations as it relied heavily on case descriptions that varied greatly in detail regarding definition of positive therapeutic response, treatment protocols, rationale for retreatment, and total number of RTX cycles, duration of follow-up and whether disease relapse occurred, and presence of adverse events. Reports with individual case data were included, but several larger series provide only statistics rather than case descriptions and could not be incorporated into our calculations. Finally, the efficacy of the studies reviewed here may not reflect a broader population-based sample of refractory non-infectious uveitis and scleritis due to referral, selection, treatment, evaluator, and/or publication bias [80].

## Conclusions

Rituximab appears to be an effective and well-tolerated option for immunosuppression in patients with non-infectious uveitis and scleritis. Authors generally favored utilizing the rheumatologic protocol and tended to report mild adverse events in about one-fifth of treated patients, with infusion reactions being most common. Uveitis associated with npAIR, JIA, VKH disease, and Behçet disease, and scleritis arising from GPA and RA all appear to be well-suited for use of RTX. In contrast, roughly half of treated patients with CAR showed a positive response. Overall, the current ophthalmologic literature strongly supports RTX use for non-infectious uveitis and scleritis refractory to corticosteroid and traditional non-corticosteroid immunosuppressive agents.

## Data Availability

The datasets used and/or analyzed during the current study are available from the corresponding author on reasonable request.

## Abbreviations

ANCA: Antineutrophil cytoplasmic antibody
CAR: Cancer associated retinopathy
ERG: Electroretinography
FDA: Food and Drug Administration
GPA: Granulomatosis with polyangiitis
HLA: Human leukocyte antigen
IgG4: Immunoglobulin G4
JIA: Juvenile idiopathic arthritis
MAR: Melanoma associated retinopathy
npAIR: non-paraneoplastic autoimmune retinopathy
RTX: Rituximab
SLE: Systemic lupus erythematosus
TNF: Tumor necrosis factor
VKH: Vogt-Koyanagi-Harada
